# Cost-effectiveness of caries excavations in different risk groups − a micro-simulation study

**DOI:** 10.1186/1472-6831-14-153

**Published:** 2014-12-15

**Authors:** Falk Schwendicke, Sebastian Paris, Michael Stolpe

**Affiliations:** Department of Operative and Preventive Dentistry, Charité−Universitätsmedizin Berlin, Aßmannshauser Str. 4-6, 14197 Berlin, Germany; Kiel Institute for the World Economy, Kiel, Germany

**Keywords:** Caries, Dental, Caries removal, Incomplete, Partial, Inequality, Caries risk, Health economics

## Abstract

**Background:**

Whilst being the most prevalent disease worldwide, dental caries is increasingly concentrated in high-risk populations. New caries treatments should therefore be evaluated not only in terms of their cost-effectiveness in individuals, but also their effects on the distribution of costs and benefits across different populations. To treat deep caries, there are currently three strategies: selective (one-step incomplete), stepwise (two-step incomplete) and complete excavation. Building on prior research that found selective excavation generally cost-effective, we compared the costs-effectiveness of different excavations in low- and high-risk patients, hypothesizing that selective excavation had greater cost-effectiveness-advantages in patients with high compared with low risk.

**Methods:**

An average tooth-level Markov-model was constructed following the posterior teeth in an initially 18-year old male individual, either with low or high risk, over his lifetime. Risk was assumed to be predicted by several parameters (oral hygiene, social position, dental service utilization), with evidence-based transition probabilities or hazard functions being adjusted for different risk status where applicable. Total lifetime treatment costs were estimated for German healthcare, with both mixed public-private and only private out-of-pocket costs being calculated. For cost-effectiveness-analysis, micro-simulations were performed and joint parameter uncertainty introduced by random sampling of probabilities. Cohort analyses were used for assessing the underlying reasons for potential differences between strategies and populations.

**Results:**

Selective excavation was more effective and less costly than both alternatives regardless of an individual’s risk. All three strategies were less effective and more costly in patients with high compared with low risk, whilst the differences between risk groups were smallest for selective excavation. Thus, the cost-effectiveness-advantages of selective excavation were more pronounced in high-risk groups, who also benefitted the most from reduced private out-of-pocket treatment costs.

**Conclusions:**

Whilst caries excavation does not tackle the underlying sources for both the development of caries lesions and the potential differences of individuals’ risk status, selective excavation seems most suitable to treat deep lesions, especially in patients with high risk, who over-proportionally benefit from the resulting health-gains and cost-savings.

## Background

Oral conditions affect 3.9 billion people globally, and untreated caries is the most prevalent disease worldwide, with an increasing concentration in high-risk populations [1, 2]. Considering the skewed prevalence of dentinal and, even more so, deep pulpo-proximal caries lesions across risk-groups [3, 4], any treatment strategy for these lesions should be evaluated not just regarding its patient-level clinical efficacy or its initial or long-term costs, but also its effect on the distribution of health benefits and costs between populations: As has been shown for other therapies, better treatments might increase the overall societal health, whilst increasing the unequal distribution of health between different populations within a society [5]. Similarly, many innovations in dentistry have been found to improve merely the health of those fortunate enough to have only limited treatment needs, whilst society generally wishes to prioritize the development of new treatments that alleviate the greater disease burden among those most at risk [1], in line with John Rawls’ theory of justice [6]. Behind a Rawlsian veil of ignorance about whether one will be in a high- or low-risk population, the theory implies that people would prioritize resources to minimize the greater expected burden of those in the high-risk population.

Deep caries lesions are usually treated by complete removal of carious dentin and restoration of the resulting cavity. This treatment often initiates a cascade of re-interventions, thereby compromising both the vitality of the pulp and the retention of the tooth [7]. Incomplete excavation of deep lesions was shown to reduce the risks of pulpal exposure and post-operative pulpal complications [8, 9], and seems suitable to delay or avoid this cascade of re-treatments. Such incomplete caries removal can be performed stepwise, with residual caries being left under a temporary excavation after the first step, followed by complete excavation in a second step. Alternatively, selective excavation can be performed, omitting the second, re-entry step and sealing the caries-affected dentin in proximity to the pulp under a definitive restoration, with an adequate seal leading to arrest and remineralization of the caries lesion [10–13]. Such selective excavation does not bear the risk of pulpal exposure at the second excavation step and was shown to reduce post-operative pulpal complications compared with two-step and complete caries removal [8, 13, 14]. Besides being clinically effective in retaining teeth and their vitality for longer, selective excavation was shown to be cost-effective as well, with significantly reduced long-term costs [15].

It remains unknown which strategy might be best suited to address the described gradient in the distribution of deep caries lesions. Demonstrating that a strategy has not only clinical advantages, but also reduces the inequality in health outcomes between groups, disrupting or weakening the link between having a high risk for developing caries and experiencing a high burden of disease and re-treatments, might be an additional argument supporting change. Model-based studies attempting such comparisons have been performed before, but are uncommon in dentistry – whilst there is a growing consensus that novel diagnostic or therapeutic strategies should also be evaluated regarding their effects on cost- and health-distributions between different risk or social groups [1, 5].

The present study analysed the cost-effectiveness of the described three excavation strategies in individuals with different risks, and explored the long-term impact of caries removal on the distribution of health. We hypothesized that selective excavation has greater cost-effectiveness-advantages over alternative treatments in patients with high compared with low risk, and additionally assessed the absolute and relative magnitude of the described cost-effectiveness differences between strategies and populations.

## Methods

### Populations, settings, perspective, time horizons and comparisons

We compared the cost-effectiveness of three interventions (selective, stepwise, complete caries removal) in the context of German healthcare, using a mixed public-private payer perspective as is characteristic for Germany. All posterior teeth in individuals from a low- or high-risk group were modelled over the lifetime of a male German patient initially aged 18 years with a remaining life expectancy of 60 years [16]. Note that “risk” was used to describe an overall result of various risk factors or predictors, i.e. oral hygiene, social position, dental service utilization, and that our study did not aim to comparatively quantify the effects of different risk factors. Risk groups were thus constructed based on the combined effects of various risk factors, with different studies using different measures to describe risk, and different cut-offs to discriminate risk groups.

### Outcomes

We evaluated the lifetime costs (in Euro) generated by initial and follow-up dental treatments. As health outcome, the average retention time of a posterior tooth (maximum: 60 years) was assessed.

### Model and assumptions

For each excavation strategy, we constructed an average tooth-level model for simulating posterior teeth. Based on the prevalence of decayed, filled or missing teeth in different risk groups (that is, caries experience), teeth were assumed to be sound, carious, filled or missing at the start of the simulation. Prevalence estimates were obtained from the best available long-term cohort study, based on data from New Zealand [17, 18], and this study was used to derive the relevant transition rates as well, which are not available for a German cohort (see below).

**Figure 1:**
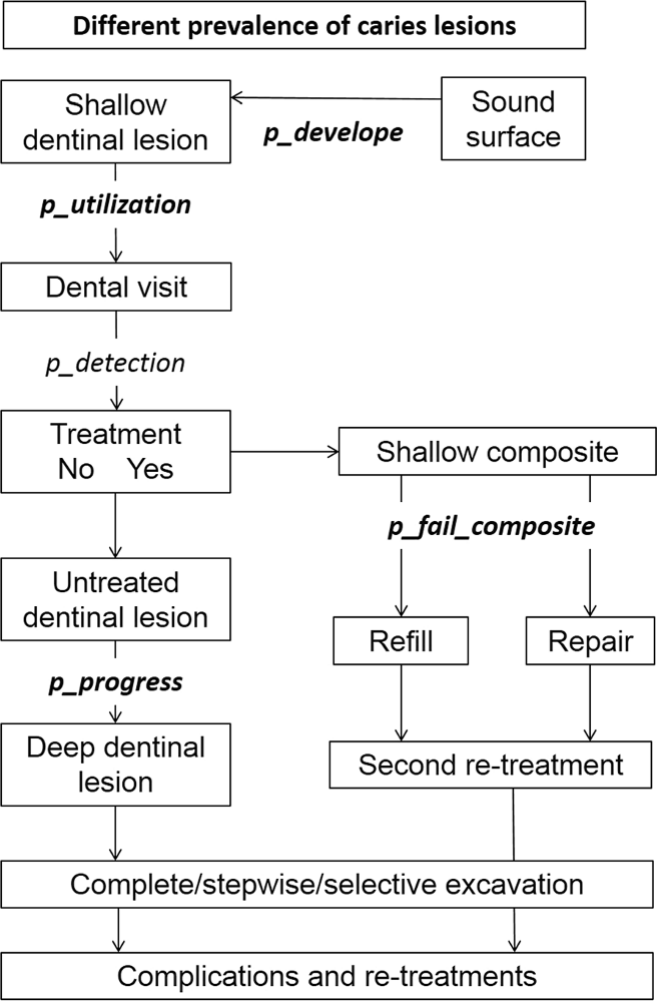
**Used Markov-model.** We followed posterior teeth in a male patient initially aged 18 years over his lifetime. The prevalence of caries lesions was assumed to differ between risk groups. The probability of a sound surface developing a shallow dentinal lesion was determined by *p_develope*. Depending on the patient’s utilization of dental services (*p_ utilization*), the patient attended for a dental checkup, where the dentists detected and invasively treated the lesion with a certain probability (*p_detection*). Treatment at this stage resulted in a shallow occlusal composite restoration, which failed according to its transition probability (*p_fail_composite*), resulting either in repair or refill according to the respective allocation probability. If failing a second time, re-treatment was assumed. Progression of a shallow lesion (according to *p_progress*) was assumed to lead to a deep dentinal lesion, which was subsequently treated by one of three caries excavation strategies. Transition probabilities in follow-up stages were modelled as described elsewhere [15]. Bold variables were found to differ according to an individual’s risk group, and were used to separately model low- and high-risk patients.

The sequence of subsequent events was constructed based on expected primary care in Germany. Depending on the attendance rate of a patient (that is, the utilization probability of available dental services), present or developing dentinal lesions could either be detected or not. Detected lesions received a two-surface adhesive restoration. Given that certain lesions might require only a single-surface restoration, we performed a sensitivity analysis to check for the impact of this assumption. Only if such lesions were not treated, they progressed to deep lesions according to progression probabilities, which were estimated separately for low- and high-risk groups. The resulting deep pulpo-proximal lesions received treatment via different caries excavation strategies and subsequent restorative treatment as soon as the patient attended. If the patient did not attend, further progression was assumed, eventually leading to the need for root-canal treatment (RCT). The model was constructed using TreeAge Pro 2013 (TreeAge, Williamstown, MA, USA). Only complications related to caries and subsequent restorations were modelled. Validation of the model (Figure 1) was performed internally, by validation against empirical data, and by an experienced health economist (MS).

### Transition probabilities

Separate transition probabilities for each risk group were extracted from the literature. We searched for evidence supporting a risk-dependent utilization of dental services, development of a dentinal caries lesion, need to re-treat directly capped pulps, composite restorations, crowns or root-canal treated teeth via screening of one electronic database (PubMed). We found evidence for risk-dependent probabilities for dental service utilization, development of dentinal caries lesions, and re-treatment of composite restorations.

Transition probabilities of sound to carious surfaces in posterior teeth (excluding third molars) were extracted from long-term data from New Zealand [17–19]. Note that we used tooth-, not surface-level data, since follow-up health states (fillings, crowns, root-canal treatments) cannot be modelled based on surfaces, but teeth. Thus, we assumed that the first carious surface – regardless if located occlusally or proximally – determined the long-term fate of the tooth. We acknowledge that this might underestimate total treatment costs, since several surfaces on one tooth might initially receive separate treatments. Data from the 10%- and 90%-trajectories were used to estimate age-dependent per-cycle hazards of developing a carious surface in low- and high-risk individuals, respectively. Transition probabilities per cycle were linearized using the exponential function and then regressed on patients’ age by means of ordinary least squares to calculate transformed age-dependent hazard functions for explorative long-term modelling beyond the reported data frame.

Transition probabilities during or after initial caries removal were based on systematic reviews [8, 14] and had been estimated previously [15]. There was no data available for estimating risk-group dependent need to re-treat differently excavated teeth. Thus, transition probabilities of selectively, stepwise or completely excavated teeth did not differ according to risk groups. Transition probabilities for follow-up stages (direct pulpal cappings, root-canal treatments, composite restorations, crowns, implant-supported crowns) have been described elsewhere [15], as have allocation probabilities. Note that the assumption of 95% of teeth with pulpal complications being root-canal treated might be desirable, but not presenting current reality [20]. We performed sensitivity analyses to check the impact of this uncertainty. Follow-up transition probabilities were risk-group adjusted only for composite restorations, using synthesized effect estimates stemming from a random-effects meta-analysis of data reported by three different studies [21–23].

### Costs, currency and discount rate

The model adopted a mixed public-private-payer perspective characteristic for German healthcare. Estimation of costs was performed as described elsewhere [15]. Briefly, calculations were based on item-fee catalogues of the statutory public and the private insurance, and standard factoring (×2.3) of chargeable item-points used to determine costs of private treatment. Total costs per course of treatment were calculated after quantification of itemized costs. We calculated total costs as well as privately covered, out-of-pocket expenses in Euro. Future costs were discounted at 3% per year [24]. No such discounting was performed for effectiveness, as a non-monetary measure was used, and interpretation of discounted retention time is not intuitional. In addition, it remains uncertain why and at what rate such discounting should be performed. Previous analyses, with which the results of this study should be compared, also did not perform effectiveness discounting [15, 25].

### Analytical methods

For each analysis, 1000 Monte-Carlo micro-simulations were performed and joint parameter uncertainty introduced by random sampling of transition probabilities and, if applicable, risk-adjustment variables from a triangular or uniform distribution of parameters between confidence intervals or ranges [26]. Simulation was performed in discrete 6-monthly cycles. Mean and standard deviations of costs (c) and effectiveness (e) as well as incremental cost-effectiveness ratios were calculated [27]. The net benefit of each excavation strategy was estimated using the formula [28]


with λ being the ceiling thresholds of willingness to pay, i.e. the additional costs a decision maker is willing to sacrifice for gaining an additional unit of effectiveness, i.e. an additional year of tooth retention [27]. The probability of a strategy to yield the highest net benefit (NB) was then calculated for λ = 0, i.e. strategies were compared only regarding their cost-difference. Moreover, we constructed cost-acceptability curves by plotting cost-effectiveness-probabilities against different ceiling thresholds. Finally, we performed sensitivity analyses to examine the impact of varying patients’ age, the used discount rate, the number of surfaces of the initial restoration and the probability of root-canal treatment or extraction being performed in case of pulpal complications.

## Results

Input-parameters for the performed simulations are summarized in Table 1, our main findings in Table 2. In patients with low risk, teeth were retained nearly life-long, with costs and effectiveness only differing minimally between strategies. Selective excavation of deep lesions was found less costly (mean lifetime treatment costs per posterior tooth were 26.91 Euro) than complete (27.80 Euro) and stepwise excavation (28.02 Euro) and similar (59.5 years average tooth retention time) or more effective than stepwise or complete (59.0 years) excavation, respectively (Table 2, upper panel). If converted to lost teeth over an individual’s lifetime, a mean of 0.13 teeth were lost if selective excavation was performed compared to 0.26 teeth for stepwise and complete excavation. In patients with high risk, these cost- and effectiveness-differences between strategies were significantly increased, with selective excavation retaining teeth longer (+1.60% or +1 year) at lower costs (up to −13.6%, or −40.71 Euro per tooth) than alternative strategies (Table 2, lower panel). If converted to the mean number of lost teeth over a lifetime, 1.3 teeth were lost if selective excavation of deep lesions was performed compared to 1.6 teeth if stepwise or complete excavation was performed.

**Table 1:** **Input variables for different risk groups**

Parameter	Variable	Disease burden low risk	Estimated from	Disease burden high risk	Estimated from
Number of decayed teeth at age 18	DT	0.01	[17]	0.11	[17]
Number of filled teeth at age 18	FT	0.08	[17]	0.54	[17]
Number of missing teeth at age 18	MT	0.01	[17]	0.01	[17]
		Probability (p) low risk		Probability (p) high risk	
Development of a dentinal caries lesion	*p_develope*	p = 0.1694e^-0.155a^	[17]	p = 198.111e^-0.414a^	[17]
No utilization of dental services	*p_utilization*	p = −0.0074a^2^ + 1.0156a + 11.318	[29]	p = −0.0282a^2^ + 2.4366a + 11.570	[29]
Progression of a lesion if untreated	*p_progress*	*p* = 0.014	[4]	p = 0.43	[4]
Failure of a composite restoration	*p_fail_composite*	p (range) = 0.0081 - 0.0094	[15]	OR (95% CI) = 2.76 (2.01-3.79)*	[21–23]

e = 2.718281828459045235.

Transition probabilities either depended on an individual’s age (a) or were constant over the lifetime. If ranges or confidence intervals were available, random sampling between those ranges or intervals was performed.

*Risk of a failing composite restoration was adjusted for high compared with low risk individuals, whilst for all other variables, separate probabilities had been calculated.

DT decayed teeth, Ft filled teeth, MT missing teeth, OR Odds Ratios.

**Table 2:** **Cost-effectiveness of different excavation strategies in individuals with different risks**

Status	Strategy	Mean (SD) tooth retention time in years	∆ (%)	Total lost teeth	Mean (SD) costs per tooth in Euro	∆ (%)	Rank (u/d)	ICER	Probability highest net-benefit (%)	Mean (SD) total private costs for all posterior teeth in Euro	∆ (%)	Probability highest net-benefit (%)
Low risk	Complete	59.0 (1)	−0.5	0.26	27.80 (12.56)	+3.3	2 (d)	−1.78	16	181.12 (64.62)	+2.8	6
	Stepwise	59.0 (1)	−0.5	0.26	28.02 (12.99)	+4.1	3 (d)	−35.12	13	176.67 (62.22)	+0.8	13
	Selective	59.5 (1)	-	0.13	26.91 (12.11)	-	1		71	175.11 (62.36)	-	81
High risk	Complete	54.0 (1)	−2.0	1.60	335.12 (22.12)	+11.8	2 (d)	−17.66	0	2233.28 (269.56)	+15.0	0
	Stepwise	54.0 (1)	−2.0	1.60	340.51 (31.95)	+13.6	3 (d)	−21.56	0	2180.32 (255.98)	+12.3	0
	Selective	56.0 (1)	-	1.33	299.80 (11.02)	-	1		100	1941.44 (242.12)	-	100

Besides absolute cost-effectiveness values (mean and standard deviations, rounded to .0/.5), differences between strategies (∆, %) were calculated relative to the highest ranked strategy. Strategies were found either dominated (more costly and less effective) or undominated (more costly, but more effective) than the highest ranked strategy.

Moreover, private out-of-pocket expenses for each strategy were calculated. ICER = incremental cost-effectiveness ratio (∆ costs/∆ effectiveness, relative to next ranked strategy). u/d = (un)dominated.

**Figure 2:**
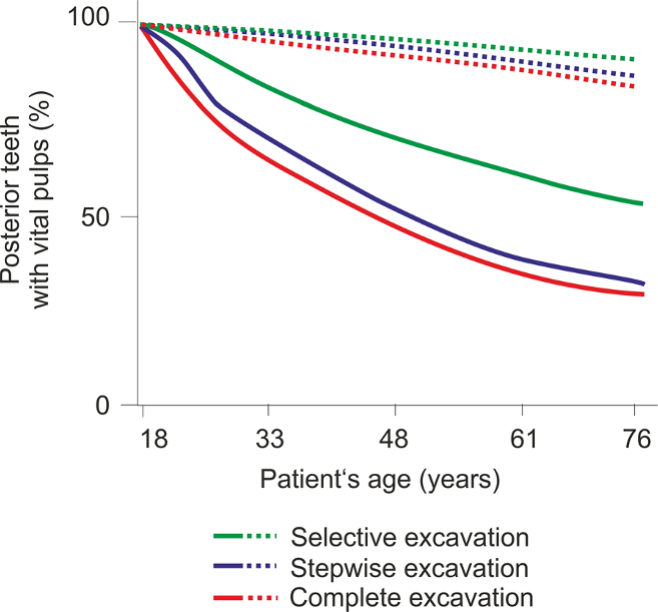
**Cohort analyses of different excavations in different risk groups.** The proportion of teeth without pulpal vitality (root-canal treated or extracted teeth) was monitored over a patient’s lifetime. Selective excavation (green, solid/dashed line: high- and low-risk individuals) retained pulpal vitality more successfully than alternative strategies (blue: stepwise, red: complete excavation), with greater advantages compared to alternative strategies in high- than low-risk individuals.

Selective excavation dominated the alternatives in the majority of simulations (Table 2). Moreover, it was also found to reduce privately covered out-of-pocket expenses of patients more strongly than alternative strategies, especially for patients at high risk, who saved up to 291.84 Euro if selective instead of complete excavation of deep lesions was performed (Table 2). The main underlying reason for the observed differences was the different retention of pulpal vitality when performing different excavations, especially in patients with high risk (Figure 2).

**Figure 3:**
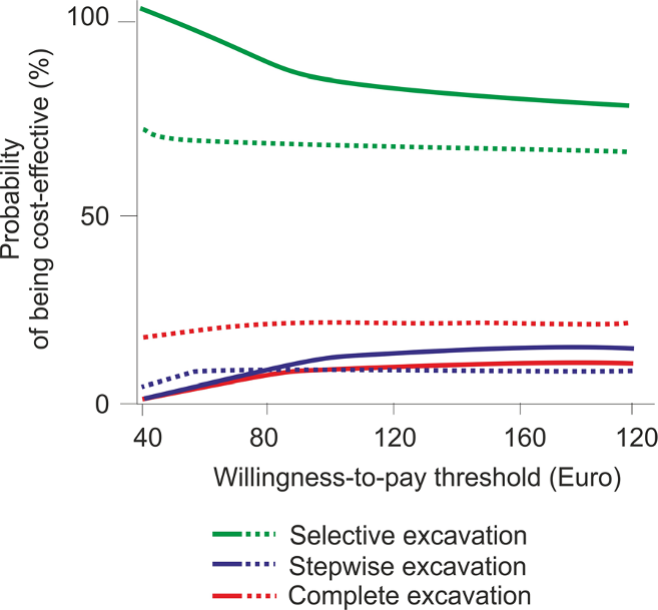
**Cost-effectiveness-acceptability curves.** The probability of a treatment being cost-effective depending on a payer’s willingness-to-pay was plotted against the maximal threshold of this willingness. With higher willingness-to-pay, cost-differences between strategies become less important for the probability of being cost-effective. Selective excavation (green, solid/dashed line: high-/low-risk individuals) had the highest probability of being cost-effective regardless of the threshold value.

Using the net-benefit approach, we found selective excavation to have the highest probability of being acceptable for a payer in terms of cost-effectiveness regardless of the payer’s willingness-to-pay threshold (Figure 3). The probability of cost-effectiveness-acceptability of selective excavation was higher for patients with high risk, and decreased with increasing willingness-to-pay, but remained >70% for all simulations and populations.

**Figure 4:**
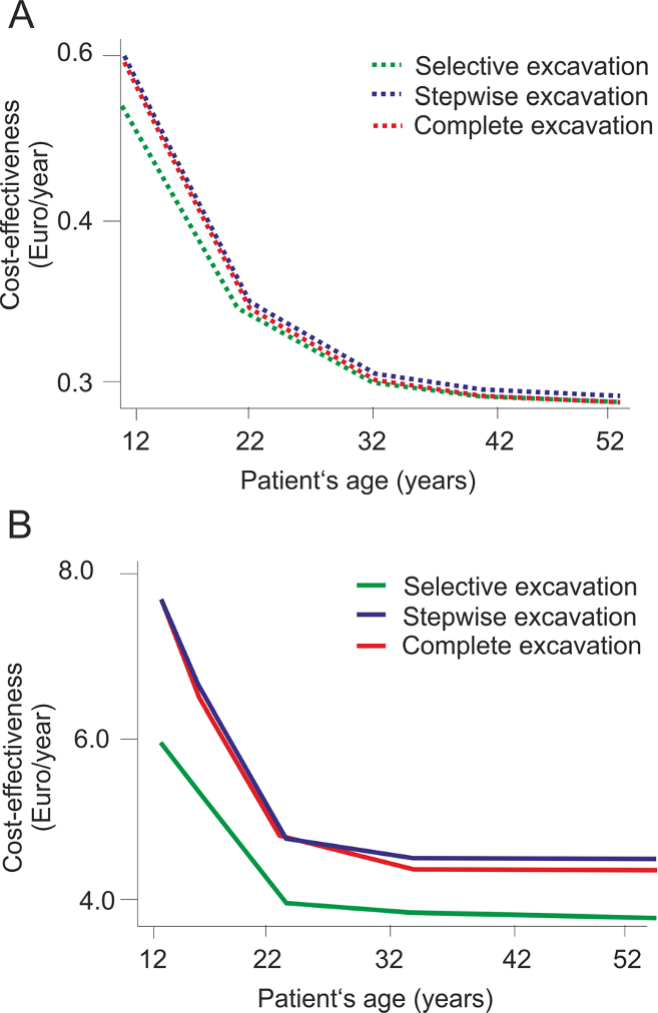
**Sensitivity analysis.** The cost-effectiveness (Euro/year) was evaluated for different strategies (green/blue/red: selective/stepwise/complete excavation) in low-risk **(A)** and high-risk **(B)** individuals depending on the initial age of a patient. Note that higher cost-effectiveness indicates higher costs per effectiveness, i.e. is less advantageous than lower cost-effectiveness. In older patients, differences between strategies were limited, especially in low-risk patients, since only a few individuals developed caries lesions and less (costly) follow-up treatments occurred. In contrast, selective excavation was most advantageous in younger patients. Cost-effectiveness was significantly worse in high- than low-risk patients.

Selective excavation had greater relative and absolute cost-effectiveness advantages compared with alternative strategies in younger than older patients (Figure 4). In patients aged >25 years, cost-effectiveness differences between strategies were minimal. Varying the discount rate between 0% and 5% per annum altered the cost-effectiveness of all strategies, but did not change the rankings of strategies. Similarly, assuming the initial restoration to be single- instead of two-surfaced only minimally changed the absolute cost-effectiveness, but not the ranking. Assuming that only 50% of teeth with pulpal complications would receive root-canal treatment and the other 50% would be extracted greatly affected the number of teeth retained over the lifetime especially in high-risk individuals: for them, 2.5, 3.5 and 3.7 would be lost over the lifetime if selective, stepwise and complete excavation were to be performed, respectively. Mean lifetime costs per tooth were affected only to a limited degree (322.12, 373.45 and 376.67 Euro, respectively). For individuals with low risk, only minimal changes compared to the base case scenario were detected (a mean of 0.3, 0.5 and 0.8 teeth were lost at mean costs per tooth of 29.95, 31.01 and 31.45 Euro, respectively).

## Discussion

There is broad evidence for a polarization of caries prevalence and experience across different populations, with a decreasing percentage of (high-risk) patients showing an increasing number of carious teeth and treatment needs [1, 4, 30]. Whilst dentistry should certainly focus on upstream approaches to tackle this problem [31] our results indicate that changing interventions more downstream could also have an impact on the resulting health outcomes, especially for those with high needs: Whilst for individuals with few lesions, low caries incidence, low progression rates and regular attendance, different strategies for treating deep lesions might not make a great difference regarding long-term tooth retention and costs – since they do not have a lot of teeth reaching the state of deep lesions –, those with many lesions, which swiftly progress and are detected only in late stages, seem to benefit over-proportionally from performing selective instead of alternative excavations. Thus, changing the caries excavation strategy might also help to break the cycle between having many lesions and experiencing a high burden of follow-up treatments. Such change would be easy, as selective instead of complete excavation does not require the dentist to acquire completely new skills or expensive additional tools. It will thus be especially suitable in settings were high-tech equipment is not be available – something to consider when discussing new treatment strategies in a global context [1].

Besides being the most effective option especially for those with high risk, selective excavation was associated with lower costs than alternative strategies, which is especially relevant in underfunded health systems with more pressing health concerns than caries and low prioritization of dental treatments. In addition, selective excavation was shown to reduce private treatment costs within the chosen healthcare setting, since expensive treatments might be either completely avoided or postponed. Such cost-saving is especially relevant for individuals with lower social status, i.e. those with high risks [32]: Cost barriers are known to decrease utilization of dental services [33], and reducing treatment costs might help increase patients’ compliance, which in turn could help improve the efficiency of dental services [34]. It should be noted that the cost-estimates in our study are typical for the German healthcare system; the issue of privately covered or reimbursed costs may differ between healthcare systems, but the general problem of cost-sensitivity in dentistry will remain.

Our results regarding lifetime tooth retention in high-risk groups seem overly optimistic compared with data from New Zealand [18], which reported 2.2 lost teeth at age 32 in the high-risk trajectory group, or data from Germany, which reported 1.5 and 4.0 lost teeth in low- and high-risk groups, respectively [30]. In our base case scenario, these numbers were not even reached over an individual’s lifetime. We have chosen the New Zealand data, as it allows modelling over a patient’s lifetime using data that supports nearly half of this life: this makes extrapolation into the lifetime frame less uncertain. No German long-term cohort data is available, and available Swedish data was not found to cover such a long time-frame [35]. It should be noted that the different health system and further socio-economic or health-related differences might influence individual behaviour; our model is thus potentially prone to distortions relative to the “true” situation in Germany.

There are several explanations for the found discrepancies between the modelled and the real-life outcomes: first, we investigated posterior teeth only, since deep caries lesions were assumed to be a problem mainly in these teeth. Thus, our numbers cannot easily be compared with reported caries experience in the whole dentition. Second, our model was based on the conservative assumptions that treatment decisions would not differ between risk groups. We have to acknowledge evidence that dentists’ decisions in identical clinical situations sometimes differ between risk groups [36] and that, generally, treatment allocations depend on patients’ characteristics [37]. Treatment decisions in patients with high risk will often be more radical, especially when faced with the option of retaining or removing posterior teeth [38]. Thus, it is all the more relevant to avoid situations where dentists are faced with the dilemma of having to choose between root-canal treatment (which is time-consuming, supposed to be painful, and expensive) and removing the tooth. We have accounted for this via sensitivity analyses and could show that by adjusting the probability of root-canal treatment instead of extraction being performed, the number of teeth retained over the lifetime decreased dramatically. We thus acknowledge that tooth retention, which we assumed to be the aim of dental treatment, may not always be the priority in dental practice. Third, our simulation did not allow for complications beyond those associated with the initial treatment, which results in an under-estimation of the overall tooth loss over patients’ lifetimes. It is however unlikely that the cost-effectiveness ranking of strategies would be affected if all possible complications were incorporated into the model.

Our conclusions must be made under the caveat of uncertainty that applies to simulation studies in general, since input parameters themselves are variable and might change in the future, and patients as well as settings are somewhat heterogeneous [39]. We have accounted for joint parameter uncertainty within our simulation, and performed additional sensitivity analyses. Nevertheless, the low number and quality of many included studies and the fact that most trials have analysed deciduous molars, whilst we modelled permanent posterior teeth, limits the reliability and generalizability of our results.

Another question is whether also the efficacy of different excavation strategies differs across risk groups: Currently, there is no indication that, for example, selective excavation performs better in patients with low than high risk, but given that lesions of different activities – which could be differently distributed between populations – are associated with different risks of pulpal complications [40], further studies should be performed to compare pulpal outcomes after different excavation strategies in populations with different risks.

The definition of risks groups was based on risk indicators, and our simulation compared risk groups by varying key variables or functions, which we had identified as factor or predictors of an individual’s risk. The “true” risk of a patient will differ, since the variables we correlated in our model do not always associate with each other. Moreover, these variables might change over a patient’s life-time, with initial treatments or changes in social status etc. possibly affecting an individual’s behaviour (oral hygiene, dental service utilization) and, subsequently, his or her risk later in life [41]. Our study evaluated the maximal reported impact of being in a certain risk group on transition probabilities, whilst it remains uncertain what the true range of effects is, depending on the analysed populations and the chosen cut-offs [32].

As mentioned, extrapolating data of short- or even midterm studies into a lifetime perspective introduces uncertainty. The effects of this uncertainty might be limited, since caries-related tooth-loss is known to happen rather earlier than later in life [42], and – in our model – discounting effects decrease the importance of costs occurring later in life. Moreover, gender and other person-specific factors, such as social position, might affect life-expectancy [43], but considering the magnitude of potential differences in life-expectancy and the demonstrated importance of early, not late caries treatments for the resulting cost-effectiveness, the impact of such heterogeneity will be limited. It remains unclear if gender differences would also affect the relative cost-effectiveness of different strategies, as caries prevalence and experience might differ between genders; no gender-specific data for deep lesions is available, though.

Needless to say, this study is based on a number of assumptions that were required because insufficient “hard” data was available. We have attempted to address the impact of these assumptions on our findings using sensitivity analyses. Additionally, the performed regression methods might have introduced bias, and future studies should consider more sophisticated methods, such as latent variable models. However, we assume this will be of limited impact, as all excavation strategies were submitted to the hazard functions calculated via these regression analyses.

## Conclusions

Within this simulation study and based on current evidence, selective excavation of deep lesions retained teeth and their vitality for longer at lower total and private out-of-pocket costs. Whilst the cost-effectiveness of all strategies decreased in patients with high compared with low risk, this decrease was smallest for selective excavation, leading to an even greater cost-effectiveness-advantage of this strategy in patients with high risk. Whilst caries excavation cannot tackle the underlying sources for the development of caries lesions and the different risks across individuals, selective excavation seems cost-effective to treat deep lesions, especially in patients with high risk, who benefit over-proportionally from the resulting health-gains and cost-savings.
